# Mastectomy with one-stage or two-stage reconstruction in breast cancer: analysis of early outcomes and patient’s satisfaction

**DOI:** 10.1007/s13304-022-01416-0

**Published:** 2022-11-19

**Authors:** Angela Gurrado, Alessandro Pasculli, Alessia Toma, Michele Maruccia, Rossella Elia, Marco Moschetta, Michele Telegrafo, Giuseppe Massimiliano De Luca, Walter Lavermicocca, Elisabetta Poli, Francesco Paolo Prete, Lucia Ilaria Sgaramella, Giuseppe Giudice, Mario Testini

**Affiliations:** 1 Department of Biomedical Sciences and Human Oncology, Academic Unit of General Surgery, University Medical School “Aldo Moro” of Bari, Policlinic Hospital of Bari, P.Zza G. Cesare 11, 70124 Bari, Italy; 2 Department of Emergency and Organ Transplantation, University Medical School “Aldo Moro” of Bari, Bari, Italy; 3 Breast Unit Policlinic Hospital of Bari, Bari, Italy

**Keywords:** Breast cancer, Skin sparing mastectomy, Nipple-sparing mastectomy, Breast reconstruction, One-stage breast reconstruction, Two-stage breast reconstruction

## Abstract

Aim of this study is to compare early post-operative outcomes and patient’s satisfaction after skin-sparing and/or nipple-sparing mastectomy (SSM/SNSM) followed either by breast reconstruction with one-stage prepectoral implantation or two-stage technique for breast cancer (BC) or BRCA1/2 mutation.

From January 2018 to December 2021, 96 patients (mean age of 51.12 ± 10.9) underwent SSM/SNSM and were divided into two groups: in group A (65 patients, 67.7%), mastectomy was followed by one-stage reconstruction; in group B (31 patients, 32.3%) by two-stage. Operative time was significantly longer in A vs. B (307.6 ± 95.7 vs. 254.4 ± 90.91; *P* < 0.05). Previous breast surgery was more common in B vs. A (29.0% vs. 7.7%; *P* < 0.05), while bilateral surgery was performed more frequently in A vs. B (40% vs. 6.5%; *P* = 0.001). All SSM/SNSM for BRCA1/2 mutation were followed by immediate prepectoral implantation. No significant differences were found between groups in terms of post-operative complications. At pathology, DCIS and invasive ST forms, such as multicentric/multifocal forms, were detected more frequently in B, while NST type in A (all *P* < 0.05). A multivariate analysis showed improved post-operative satisfaction at BREAST-Q survey in Group A (*P* = 0.001). Encouraging oncologic outcomes after SSM/SNSM for BC enabled the improvement of breast reconstructive techniques. One-stage reconstruction is characterized by better aesthetic outcomes and by greater patient’s satisfaction. When SSM/SNSM is technically difficult to perform, as in multicentric/multifocal forms or previous breast surgery, mastectomy followed by two-stage reconstruction should be considered to achieve a radical surgery.

**Supplementary Information:**

The online version contains supplementary material available at 10.1007/s13304-022-01416-0.

## Introduction

Breast cancer (BC) is the most common cancer in women in the European Union and a rare cancer in men. The estimated incidence in Europe is 416.000 cases in 2018, while in Italy is 55.000 in 2020 [1]. Overall, 5-year survival is 82% in Europe, 87% in Italy [1].

The choice of the treatment is based on the tumour’s histological and morphological characteristics. Patients with locally advanced or inflammatory BC may undergo neoadjuvant chemotherapy (NAC) followed by surgery, while patients with small ductal carcinoma in situ (DCIS) and invasive BC may undergo breast-conserving surgery (BCS) followed by breast irradiation. For widespread DCIS, invasive breast cancer or in male patients, mastectomy should be considered. Overall, skin-sparing and/or nipple-sparing mastectomy (SSM and/or SNSM) is performed in about 44–55% of cases, and it has an important psychological impact, so many patients choose breast reconstruction when available [2–5]. The wide popularity of SSM and/or SNSM is supported by oncological safety that appears equal to total mastectomies [4–6].

Currently, breast reconstructive techniques are divided into autologous reconstruction with flaps [transverse rectus abdominis (TRAM), deep inferior epigastric perforators (DIEP), and others] and heterologous reconstruction (prosthesis-based techniques), in one or in two stages [2–5]. An implant-based breast reconstruction method is used after approximatively 80% of mastectomies [7]. The SSM and/or SNSM preserves the natural skip flap and adequate volume, allowing one-stage technique by immediate reconstruction with permanent breast implant; the implant is usually enclosed by an acellular dermal matrix, in a prepectoral pocket [2–5, 8–15]. The one-stage reconstruction technique needs only one operative time and consequently less anaesthesia time, fewer recovery days, and follow-up visits, so the economic costs are lower than the two-stage reconstruction. Patients report a better psychological well-being, although the one-stage technique could see a twofold increase of risk of implant loss, of post-operative complications and re-surgery [2–5, 16].

Generally, the two-stage technique involves positioning of a tissue expander in a retro-pectoral pocket after mastectomy, followed by exchange with an implant in a second stage [2–5, 17]. Although the two-stage reconstruction may heal more easily, it has several disadvantages: difficulty to achieve lower pole projection and ptosis; it needs a second operative time, so patients suffer from psychological distress, given also the sensation of foreign body and chronic pain. It also has larger economic impact in terms of global hospitalization costs [2–5, 16].

Typically, selection criteria for choosing reconstruction technique are divided into patient’s characteristics (age, familial history, general health status, and patient’s wishes) and tumour’s features (localization, size, lymph-nodal state, and receptor state). Moreover, the prerequisites for the one-stage reconstruction are: an SSM and/or SNSM and a BMI between 25 and 30 kg/m^2^ [2–5, 16].

Existing literature has analysed pros and cons of one-stage and two-stage reconstruction, but it appears that more studies are needed to draw solid conclusions [2–5, 16, 18–20]

Aim of this retrospective cohort study is to compare SSM and/or SNSM for BC or BRCA1/2 mutation with breast reconstruction by one-stage technique to two-stage technique in terms of post-operative outcomes and patient’s satisfaction.

## Materials and methods

For this study, patients who underwent mastectomy with breast reconstruction for BC or diagnosis of BRCA 1/2 mutation were selected and divided in Group A (one-stage reconstruction) and Group B (two-stage reconstruction). They were selected from the database of our Academic Unit of General Surgery from January 2018 to December 2021.

After diagnosis of BC, supported by ultrasound, mammography, and needle biopsy on suspicious lesions, the multidisciplinary team of Breast Care Unit of Policlinic of Bari provided each patient with the information they need about surgery, pros and cons of the surgical technique, and possible post-operative outcomes. All patients were followed up by the multidisciplinary team.

A protocol was registered prior to conducting the study, which was approved by the Independent Ethical Committee of the University of Bari (Protocol 2020/6422) and was performed in accordance with the Declaration of Helsinki of 1975, revised in 2013. All patients gave their informed written consent before participating in the study. This work has been reported in line with the Strobe guidelines [21].

### Patient’s selection

Inclusion criteria for the study were SSM and/or SNSM for BC or risk-reducing surgery and either one-stage or two-stage breast reconstruction, minimum follow-up time of 6 months. Exclusion criteria included: stage IV disease; patients considered at high risk of recurrence; patients who underwent BCS; mastectomy without reconstruction; reconstruction with DIEP flap; patients who declined to have the prepectoral procedure (they had traditional breast reconstruction techniques). Allocation to either one-stage or two-stage breast reconstruction were based on BMI: BMI < 25 has been considered an indication to two-stage reconstruction.

### Surgical technique

SSM and/or SNSM was performed using peri-areolar or inframammary incisions, with the choice of incision line usually depending on breast size and distance from the inframammary fold to the clavicle level. All glandular tissue was removed, keeping the subcutaneous fat layer sufficiently thick (≥ 3 mm) to preserve the blood supply through the sub-dermal vascular plexus. When fresh-frozen retroareolar biopsy results were negative, the nipple–areola complex was preserved.

In one-stage reconstruction, provided that the sub-dermal plexus and [4, 5, 22] the skin flap has an adequate thickness, an implant (MENTOR CPG™ Gel Breast Implants with Cohesive III™, Mentor, Santa Barbara, CA, USA) covered by an acellular dermal matrix (ADM) was placed in a prepectoral pocket. A pre-shaped, 0.6-mm-thick, porcine, noncross-linked ADM Braxon^®^ (MBP Biologics, Neustadt-Glewe, Germany, license holder Decomed, Marcon, Venezia, Italy) was used in our study, and it is fixated to the pectoralis major muscle with absorbable sutures.

In two-stage breast reconstruction after SSM and/or SNSM, a tissue expander (MENTOR CPX^™^4 Breast Tissue Expander, Mentor, Santa Barbara, CA, USA) was placed in a retromuscular pocket, created by suturing the pectoralis major to the serratus anterior. The size of the expander is chosen based on its base diameter and volume. The expander is filled with approximately 50–100 cc of saline and 2 cc of methylene blue [2, 4, 5] and expanded weekly or biweekly until its final volume; 4–6 weeks after total expansion or the end of adjuvant therapy, an implant exchange is performed.

Two suction drains (Blake’s size 10) were inserted in the subcutaneous and axillary pockets if lymph-node dissection was performed.

Perioperative antibiotics (teicoplanin 400 mg) were given 30 min before the surgical incision and repeated after 12 h; the one-stage group continued antibiotic once daily for 5 days postoperatively.

### Data collection and outcome evaluation

Demographic data, pre-operative diagnosis, type of mastectomy and reconstruction, operative time, use and duration of drain, length of hospitalization, post-operative histology, and post-operative complications were retrieved from our prospectively designed database.

Post-operative outcomes were represented by: surgical site infection (SSI), defined as culture-proven infection and/or removal of the implant without immediate replacement as per the Centers of Disease Controlled and Prevention (CDC) guidelines for SSI [23]; early and late seroma, defined as palpable fluid collection on clinical examination with or without imaging confirmation; skin flap necrosis; wound dehiscence; hematoma; “red breast syndrome”; rippling and implant extrusion. Post-operative complications were ranked according to the Clavien–Dindo classification [24]. The rates of unplanned readmissions, unplanned re-surgery, and rates of local and distant recurrence were also recorded during follow-up time.

Patient’s satisfaction was evaluated using the BREAST-Q survey [20] to quantify post-operative satisfaction, in terms of pain and aesthetics, minimum 6 months after surgery. Minimum follow-up time was 6 months.

### Study size

Study size was determined upon power calculation on the endpoint “surgical complications” used in a randomized study [25]. According to the type of reconstruction that patients underwent, 65 patients were allocated in group A (one-stage reconstruction) and 31 in group B (two-stage reconstruction).

### Statistical analysis

Chi-square and Fisher’s exact (for ordinal variables) and Student’s *t* (for continuous variables) tests were used to compare group A (mastectomy followed by one-stage reconstruction) to group B (mastectomy followed by two-stage reconstruction). A *p* value less than 0.05 was considered statistically significant.

The scores of BREAST-Q survey were displayed onto a 0–100 scale, with higher scores corresponding to increased patients’ satisfaction [20, 22].

A multivariate analysis was performed using ordered logistic regression, to relate significantly different outcomes with a model including type of reconstruction along with age, BMI, ASA, previous breast surgery, previous neck/chest radiotherapy, comorbidities, NAC, bilateral/monolateral surgery, axillary dissection, nipple-areolar preservation, overall morbidity, histology, and multicentric/multifocal disease.

## Results

From January 2018 to December 2021, 241 patients underwent breast surgery at our Academic Unit of General Surgery. According to the exclusion criteria, 145 (60.1%) patients were not included in the study: stage IV disease (*N* = 1; 0.4%); patients deemed at high risk of recurrence (*N* = 1; 0.4%); BCS (*N* = 96; 39.8%); mastectomies without reconstruction (*N* = 30; 12.4%); mastectomies with DIEP flap reconstruction (*N* = 15; 6.2%). In addition, 1 (0.4%) patient was excluded for use of drugs and 1 (0.4%) patient was not included because of missing data.

The study population consisted of 96 patients (mean age of 51.12 ± 10.9, range: 29–81 years) and 124 operated breasts; 65 patients underwent SSM and/or SNSM followed by one-stage reconstruction (67.7%; group A), and 31 patients by two-stage reconstruction (32.3%; group B).

Table 1 shows demographic data, pre-operative histology, type of surgery, and clinical characteristics of the patients.

**Table 1:** Demographic data, pre-operative histology, type of surgery, and clinical characteristics

	Group A (*N* = 65)	Group B (*N* = 31)	P*
Age (years)	52.1 ± 11.1	49.1 ± 10.6	0.210
BMI^1^ (kg/m^2^)	25.1 ± 5.2	23.6 ± 3.6	0.177
Pre-operative histology N (%)			
DCIS	9 (13.8)	9 (29.0)	**0.004**
Invasive			
NST	35 (53.8)	14 (45.2)	
Lobular	7 (10.8)	6 (19.4)	
Tubular	0 (0.0)	3 (9.7)	
Mixed	3 (4.6)	1 (3.2)	
Mutation BRCA 1	5 (7.7)	0 (0.0)	
Mutation BRCA 2	6 (9.2)	0 (0.0)	
ASA^2^ N (%)			
1	32 (49.2)	16 (51.6)	1.000
2	26 (40.0)	12 (38.7)	
3	6 (9.2)	3 (9.7)	
Previous breast surgery	5 (7.7)	9 (29.0)	**0.011**
Previous neck/chest radiotherapy	2 (3.1)	3 (9.7)	0.167
Comorbidities N (%)	34 (52.3)	13 (41.9)	0.387
Hypertension	15 (23.1)	5 (16.1)	0.593
Diabetes	1 (1.5)	1 (3.2)	0.544
Asthma	5 (7.7)	1 (3.2)	0.660
Smoking	10 (15.4)	5 (16.2)	1.000
Alcohol	1 (1.5)	0 (0)	1.000
Other types of cancer	7 (10.8)	3 (9.7)	1.000
Anticoagulant/antiplatelet drugs	1 (1.5)	0 (0)	1.000
Corticosteroids/Immunosuppressive drugs	3 (4.6)	0 (0)	0.549
NAC	3 (4.6)	3 (9.7)	0.384

Data shown as average ± standard deviation or as absolute frequency with percentage in brackets. *Student’s *t* test for independent samples for continuous values or χ^2^/Fisher’s exact test for ordinal/binomial variables. *Group A* one-stage reconstruction, *Group B* two-stage reconstruction. Bold emphasized values are statistically significant

*NAC* neoadjuvant chemotherapy

^1^Body mass index

^2^American Society of Anesthesiologists

No significant differences were found in terms of age, BMI, ASA score, comorbidity, previous neck/chest radiation therapy, and NAC. In terms of pre-operative histology, DCIS and the invasive ST forms were more frequently detected in group B (29 and 32.3%, respectively) vs. A (13.8 and 15.4%, respectively), while invasive NST type rate was higher in A (53.8%) vs. B (45.2%) (all *P* < 0.05). All patients affected by mutation BRCA 1 and/or 2 underwent SSM and/or SNSM with immediate prepectoral implantation. Previous breast surgery was significantly more common in the group B (29.0%) vs. A (7.7%; *P* < 0.05).

The perioperative data are summarized in Table 2. Previous bilateral surgery was significantly more represented in group A (40%) vs. B (6.5%) and in terms of monolateral operation in B (93.5%) vs. A (60%) (all *P* = 0.001). Operative time was significantly greater (*P* < 0.05) in group A (307.6 ± 95.7) compared with B (254.4 ± 90.91).

**Table 2:** Perioperative data

Perioperative data, *N* (%)	Group A (*N* = 65)	Group B (*N* = 31)	P*
Bilateral surgery	26 (40.0)	2 (6.5)	**0.001**
Monolateral surgery	39 (60.0)	29 (93.5)	
Sentinel lymph-node biopsy	50 (76.9)	27 (87.1)	0.286
Axillary dissection	19 (29.2)	14 (45.2)	0.168
Surgical enlargement	20 (30.8)	11 (35.5)	0.648
Nipple–areolar complex removal	33 (50.8)	15 (48.4)	0.829
Operative time (min)	307.6 ± 95.7	254.4 ± 90.91	**0.012**
Hospital stay (hours)	117.4 ± 32.5	110.7 ± 28.2	0.332
Time to drain removal (days)	14.4 ± 8.7	15.2 ± 11.51^1^	0.683
Adjuvant therapy after surgery	26 (40.0)	15 (48.4)	0.510

Data shown as absolute frequency with percentage in brackets. *χ^2^/Fisher’s exact test for ordinal/binomial variables. *Group A* one-stage reconstruction, *Group B* two-stage reconstruction. Bold emphasized values are statistically significant

^1^Referred to first-stage surgery

No mortality occurred. Overall post-operative morbidity was 23.9%, and Table 3 shows detailed post-operative complications. No statistically significant differences were found between the groups in terms of post-operative morbidity.

**Table 3:** Post-operative complications

Complications, *N* (%)	Group A (*N* = 65)	Group B (*N* = 31)	P*
Nil	46 (70.8)	27 (87.1)	0.124
Surgical site infection	1 (1.5)	0 (0)	1.000
Early seroma	1 (1.5)	0 (0)	1.000
Late seroma	1 (1.5)	0 (0)	1.000
Skin flap necrosis	3 (4.6)	0 (0)	0.549
Wound dehiscence	1 (1.5)	0 (0)	1.000
Nipple necrosis	5 (7.7)	0 (0)	0.174
partial	2 (3.1)	0 (0)	1.000
complete	3 (4.6)	0 (0)	0.549
Haemorrhage	1 (1.5)	1 (3.2)	0.544
Red breast syndrome	0 (0)	0 (0)	–
Capsule contracture	0 (0)	0 (0)	–
Rippling	0 (0)	0 (0)	–
Implant loss	1 (1.5)	–	–
Re-surgery	4 (6.2)	3 (9.7)	0.678
Conversion to two stages	1 (1.5)	–	–

Clavien–Dindo classification. Data shown as average ± standard deviation or as absolute frequency with percentage in brackets. *Group A* one-stage reconstruction, *Group B* two-stage reconstruction. *Student’s *t* test for independent samples for continuous values or χ^2^/Fisher’s exact test for ordinal/binomial variables

^1^Referred to first-stage surgery

At post-operative histology (Table 4), DCIS (22.5%) and invasive ST types (29%), as well as the multicentric or multifocal forms (64.5%), were significantly more common in mastectomies of the group B vs. A (13.8, 19.9, and 35.4%, respectively; all *P* < 0.05), whereas the invasive NST (49.2%), and the absence of malignancy (15.4%) (i.e., prophylactic surgery for BCRA 1–2) were more detected in each ones of A vs. B (48.4% and 0%, respectively; all *P* < 0.05).

**Table 4:** Histology

Histology *N* (%)	Group A (*N* = 65)	Group B (*N* = 31)	P*
DCIS	9 (13.8)	7 (22.5)	**0.036**
Invasive			
NST	32 (49.2)	15 (48.4)	
Lobular	9 (13.8)	4 (12.9)	
Tubular	1 (1.5)	4 (12.9)	
Mixed	3 (4.6)	1 (3.2)	
Benign	10 (15.4)	0 (0)	
PcR	1 (1.5)	0 (0)	1.000
Grading			
G1	3 (4.6)	4 (12.9)	0.382
G2	17 (26.1)	10 (32.2)	
G3	31 (47.7)	14 (45.1)	
Endolymphatic invasion	10 (15.3)	2 (6.5)	0.200
Endovascular invasion	18 (27.7)	5 (16.1)	0.133
Estrogen receptor	39 (60.0)	25 (80.5)	0.553
Progesterone receptor	32 (49.2)	18 (58.1)	1.000
Her2			
0	20 (30.7)	9 (29.0)	0.380
1 +	19 (29.2)	9 (29.0)	
2 +	4 (6.2)	5 (16.1)	
3 +	2 (3.1)	3 (11.5)	
Ki67	18.4 ± 14.2	19.4 ± 17.5	0.791
Positive deep margin	4 (6.2)	3 (9.7)	0.681
Multicentric/multifocal	23 (35.4)	20 (64.5)	**0.016**

Data shown as average ± standard deviation or as absolute frequency with percentage in brackets. *Group A* one-stage reconstruction, *Group B* two-stage reconstruction. *PcR* Pathological complete response after neoadjuvant *Student’s *t* test for independent samples for continuous values or χ^2^/Fisher’s exact test for ordinal/binomial variables. Bold emphasized values are statistically significant

Table 5 accounts for patients’ psychological wellness and aesthetic satisfaction, calculated through BREAST-Q survey. All the patients adequately filled the five domains of the questionnaire and were included in the analysis. Patients in group A scored statistically higher level of satisfaction with the implant and of post-operative satisfaction in comparison with each ones of group B (*P* < 0.05).

**Table 5:** Breast Q evaluation

	Group A (*N* = 65)	Group B (*N* = 31)	P*
Psychosocial well-being	79.3 ± 25.5	69.1 ± 26.7	0.078
Pre-operative satisfaction	86.6 ± 20.3	83.6 ± 21.4	0.521
Post-operative satisfaction	82.4 ± 24.9	56.4 ± 21.2	**0.000**
Satisfaction with the implant	92.5 ± 19.1	79.6 ± 27.8	**0.014**
Chest pain	32.2 ± 33.5	46.6 ± 39.0	0.078
Adverse effects after radiotherapy	17.2 ± 33.2	16.6 ± 26.6	0.950
Pain expectations	49.6 ± 45.4	53.5 ± 46.5	0.732
Aesthetic expectations	62.8 ± 42.8	62.0 ± 41.4	0.939

Data shown as average ± standard deviation. *Group A* one-stage reconstruction, *Group B* two-stage reconstruction

*Student’s *t* test for independent samples

A multivariate analysis with ordered logistic regression confirmed the significance of the differences between group A and group B for what concerns post-operative satisfaction (*P* = 0.001), after accounting for age, BMI, ASA, previous breast surgery, previous neck/chest radiotherapy, comorbidities, NAC, bilateral/monolateral surgery, axillary dissection, nipple-areolar preservation, overall morbidity, histology, and multicentric/multifocal disease (Table 6).

**Table 6:** Multivariate analysis for post-operative satisfaction

	Coef	Std. Err	z	P*	95% Conf. interval	
Type of reconstruction	− 1.87	0.57	− 3.26	**0.001**	− 2.99	− 0.75
Age	0.01	0.02	0.54	0.588	− 0.03	0.06
BMI^1^	− 0.03	0.06	− 0.46	0.643	− 0.14	0.09
ASA^2^	0.12	0.57	0.21	0.833	− 0.99	1.23
Previous breast surgery	− 0.07	0.81	− 0.09	0.928	− 1.66	1.51
Previous neck/chest radiotherapy	− 3.63	1.21	− 2.98	**0.003**	− 6.02	− 1.24
Comorbidities	− 0.40	0.55	− 0.72	0.47	− 1.48	0.69
NAC^3^	− 0.47	1.17	− 0.40	0.691	− 2.75	1.82
Bilateral/monolateral surgery	− 0.68	0.54	− 1.26	0.207	− 1.74	0.38
Axillary dissection	− 0.72	0.52	− 1.40	0.161	− 1.73	0.29
Nipple-areolar complex removal	0.02	0.52	0.04	0.964	− 1.00	1.05
Overall morbidity	1.00	0.57	1.75	0.081	− 0.12	2.11
Post-operative histology	− 0.12	0.16	−0.71	0.476	− 0.44	0.20
Multicentric/multifocal	− 0.94	0.53	−1.77	0.076	− 1.99	0.10

*NAC* neoadjuvant chemotherapy

^1^Body mass index

^2^American Society of Anesthesiologists

*Ordered logistic regression model

## Discussion

The study aims to evaluate the early outcomes after SSM and /or SNSM for BC or diagnosis of BRCA1/2 mutation followed by breast reconstruction with one- or two-stage technique. Clinical studies have demonstrated that performing immediate implant-based breast reconstruction after SSM and /or SNSM does not impact on post-operative recurrence, survival, or delayed diagnosis of recurrence [4, 5, 7].

Nevertheless, the choice of BCS is preferred when possible, and the mastectomy is indicated in all cases of widespread DCIS or invasive BC when the clear margins have not been guaranteed and in cases of BCRA1/2 mutations. To improve the psychological well-being of the patients, SSM and/or SNSM are increasingly followed by one-stage breast reconstruction. Despite the higher risk of post-operative complications [2, 14], patients had major aesthetic satisfaction and psychological well-being [4, 5, 19], also because they did not need a second operative time and a longer recovery. However, the two-stage reconstruction remains the gold standard for selected cases, thanks to its easy of healing [2, 16].

Each of these techniques has pros and cons, which depend also on patient’s and tumour’s features. Therefore, in this study, post-operative and aesthetic outcomes are compared between patients who underwent one-stage reconstruction and patients who underwent two-stage reconstruction after SSM and/or SNSM.

This study has some limitations, because it is retrospectively designed and performed in a single institution; however, a few previous studies have been designed like this in literature. Another limitation is the different distribution of patients in the two groups according to their diagnosis.

No significative difference was found between the two groups in terms of demographic and pre-operative features. Although BMI had no significative differences between the two groups, it was important for patients’ selection. A patient with a BMI between 25 and 30 could undergo mastectomy followed by one-stage reconstruction, because values out of the range lead to major complications, like nipple–areola complex or flap necrosis and bad aesthetic outcomes. In our study, BMI is higher in group A than B, even if without statistical significance, because proper the adequate thickness of subcutaneous fat layer is discriminant to perform the prepectoral implantation.

Contrary to previous reports [26, 27], our study showed that 29% of patients who underwent tissue expander reconstruction also underwent a previous breast surgery, while only 7.7% of patients who underwent one-stage reconstruction had a history of breast surgery (*P* < 0.05). The explanation could probably be linked to the surgical complexity correlated to preserve an adequate thickness of subcutaneous flap due to previous breast scar. Moreover, patients with ipsilateral loco-regional recurrence after BCS plus radiotherapy are at higher risk of complications if underwent SSM and/or SNSM and immediate direct-to-implant technique [7, 28].

The distribution of bilateral and monolateral surgery showed statistical differences between the groups, and we hypothesized that the risk-reducing mastectomies, often bilateral, impacted on the higher frequency in immediate reconstruction group.

Operative time, such as reported in the previous study of Roostaeian et al. [29], significantly increased in SSM and/or SNSM undergoing immediate prepectoral implantation. This difference is likely due to meticulous dissection of the layers representing a technical challenging, although in this study, it is also related to the higher bilateral/monolateral ratio in the immediate reconstruction group.

At post-operative histology, the pre-operative statistical difference was confirmed, and the DCIS and the invasive ST forms were treated more commonly with SSM and/or SNSM followed by two-stage reconstruction. On the contrary, the invasive NST forms and all the cases of absence of neoplastic lesions, such as risk-reducing mastectomies and pathological complete response after NAC, benefited from the one-step operation. The reasons of this distribution are not clarified, but could be represented by the high incidence of multicentric/multifocal forms in our series. Often, a multicentric/multifocal cancer, especially ST forms, may affect the cutaneous or subcutaneous tissue, so surgeons must remove that affected skin area and this makes difficult or impossible to insert a permanent implant in one stage. In the previous literature, several possibilities are described: there is not a better approach of all [25], but the choice is subjected to patient’s and tumour’s features [25, 30]. In case of breast cancer in a patient with BRCA1/2 mutation, for example, for the contralateral reconstruction after prophylactic mastectomy, the technique depends on the possibility of post-operative radiation therapy [31].

A meta-analysis on 16 studies [16] found several significative differences between the two techniques in terms of post-operative outcomes. In mastectomies followed by one-stage reconstruction, post-operative complications were higher than two-stage operation: in particular, the risks for implant loss and for major medical complications like pneumonia [18, 32], or skin necrosis and infection [18, 32]. Instead, in our analysis, such as another meta-analysis [34] there were not any significant differences between the two groups [33]. The explanation could be that the surgical skill and the team learning curve based on patient selection, surgical judgement, and perioperative management play a fundamental role in terms of post-operative morbidity.

Using the BREAST-Q survey, the patients who underwent mastectomy followed by one-stage reconstruction reported a significantly higher post-operative satisfaction and satisfaction with the implant than patients who underwent two-stage reconstruction, because of more natural aesthetic outcomes, although the difference in terms of better psychosocial well-being was not statistically significant. Similarly, the patients in group A suffered less post-operative chest pain than patients in B group, probably due to the lack of muscular distress. In previous literature, mixed results are described: Susarla et al*.* [35] considered the two techniques comparable in terms of post-operative satisfaction, while Roosteaian et al*.* [29] confirmed that patients are more satisfied by one-stage reconstruction. As already discussed, the distribution of patients according to their diagnosis was different among the two groups; anyway, the significant difference of post-operative satisfaction was maintained applying the ordered logistic regression model.

## Conclusions

The oncologic outcomes after multimodal treatment of the breast cancer are encouraging, and these data are allowed to focus also on the reconstructive aspects for the global well-being of the woman.

Both one-stage technique and two-stage techniques are good post-mastectomy reconstructing treatments. One-stage reconstruction is characterized by better aesthetic outcomes and by greater patient’s satisfaction. There were no significant differences between the two groups in terms of pre-operative features, but BMI should be considered to avoid possible complications associated with high or low body mass index.

In patients who underwent previous breast surgery or with a diagnosis of multicentric/multifocal breast cancer, the two-stage reconstruction should be considered, because of the difficulty to perform a safe SSM and/or SNSM.

For prophylactic treatments, in which SSM and /or SNSM are performed, one-stage reconstruction with the implantation of a permanent implant should be considered a gold standard, for a higher emphasis on cosmesis.

However, the appropriate patient’s selection and the radical surgery of the breast cancer should be prioritized for oncologic purpose over aesthetic need.

Further studies are needed to improve the assessment of long-term oncological outcomes and to standardize the use of these two techniques in the surgical practice.

## Supplementary Information

Below is the link to the electronic supplementary material.

Supplementary file1 (XLSX 49 KB)

## Data Availability

All data generated or analysed during this study are included in this published article (and its additional files).
