# Correction to: Mastectomy with one-stage or two-stage reconstruction in breast cancer: analysis of early outcomes and patient’s satisfaction

**DOI:** 10.1007/s13304-022-01443-x

**Published:** 2022-12-17

**Authors:** Angela Gurrado, Alessandro Pasculli, Alessia Toma, Michele Maruccia, Rossella Elia, Marco Moschetta, Michele Telegrafo, Giuseppe Massimiliano De Luca, Walter Lavermicocca, Elisabetta Poli, Francesco Paolo Prete, Lucia Ilaria Sgaramella, Giuseppe Giudice, Mario Testini

**Affiliations:** 1 Department of Biomedical Sciences and Human Oncology, Academic Unit of General Surgery, University Medical School “Aldo Moro” of Bari, Policlinic Hospital of Bari, P.Zza G. Cesare 11, 70124 Bari, Italy; 2 Department of Emergency and Organ Transplantation, University Medical School “Aldo Moro” of Bari, Bari, Italy; 3 Breast Unit Policlinic Hospital of Bari, Bari, Italy

**Correction to: Updates in Surgery** 10.1007/s13304-022-01416-0

The family names and given names were incorrect in the published article.

Family Name: Gurrado - Given Name: Angela, Family Name: Pasculli - Given Name: Alessandro, Family Name: Toma - Given Name: Alessia, Family Name: Maruccia - Given Name: Michele, Family Name: Elia - Given Name: Rossella, Family Name: Moschetta - Given Name: Marco, Family Name: Telegrafo - Given Name: Michele, Family Name: De Luca - Given Name: Giuseppe Massimiliano, Family Name: Lavermicocca - Given Name: Walter, Family Name: Poli - Given Name: Elisabetta, Family Name: Prete - Given Name: Francesco Paolo, Family Name: Sgaramella - Given Name: Lucia Ilaria, Family Name: Giudice - Given Name: Giuseppe, Family Name: Testini - Given Name: Mario.

The original article has been updated.

